# Engineering Induced Pluripotent Stem Cells for Cancer Immunotherapy

**DOI:** 10.3390/cancers14092266

**Published:** 2022-05-01

**Authors:** Yang Zhou, Miao Li, Kuangyi Zhou, James Brown, Tasha Tsao, Xinjian Cen, Tiffany Husman, Aarushi Bajpai, Zachary Spencer Dunn, Lili Yang

**Affiliations:** 1 Department of Microbiology, Immunology & Molecular Genetics, University of California, Los Angeles, CA 90095, USA; zzydcat@g.ucla.edu (Y.Z.); ericmli0507@g.ucla.edu (M.L.); kuangyizhou@g.ucla.edu (K.Z.); brownjimw0@gmail.com (J.B.); tytsao@g.ucla.edu (T.T.); xicen@g.ucla.edu (X.C.); tiffanyhusman@g.ucla.edu (T.H.); abajpai2023@g.ucla.edu (A.B.); zacharsd@usc.edu (Z.S.D.); 2 Mork Family Department of Chemical Engineering and Materials Science, University of Southern California, Los Angeles, CA 90089, USA; 3 Eli and Edythe Broad Center of Regenerative Medicine and Stem Cell Research, University of California, Los Angeles, CA 90095, USA; 4 Jonsson Comprehensive Cancer Center, David Geffen School of Medicine, University of California, Los Angeles, CA 90095, USA

**Keywords:** induced pluripotent stem cell (iPSC), immunotherapy, cancer, allogeneic, off-the-shelf, reprogramming, chimeric antigen receptor (CAR), T, natural killer (NK), invariant natural killer T (iNKT), gamma delta T (γδ T), mucosal-associated invariant T (MAIT), macrophages (Mφs)

## Abstract

**Simple Summary:**

Induced pluripotent stem cells (iPSCs) that can be genetically engineered and differentiated into different types of immune cells, providing an unlimited resource for developing off-the-shelf cell therapies. Here, we present a comprehensive review that describes the current stages of iPSC-based cell therapies, including iPSC-derived T, nature killer (NK), invariant natural killer T (iNKT), gamma delta T (γδ T), mucosal-associated invariant T (MAIT) cells, and macrophages (Mφs).

**Abstract:**

Cell-based immunotherapy, such as chimeric antigen receptor (CAR) T cell therapy, has revolutionized the treatment of hematological malignancies, especially in patients who are refractory to other therapies. However, there are critical obstacles that hinder the widespread clinical applications of current autologous therapies, such as high cost, challenging large-scale manufacturing, and inaccessibility to the therapy for lymphopenia patients. Therefore, it is in great demand to generate the universal off-the-shelf cell products with significant scalability. Human induced pluripotent stem cells (iPSCs) provide an “unlimited supply” for cell therapy because of their unique self-renewal properties and the capacity to be genetically engineered. iPSCs can be differentiated into different immune cells, such as T cells, natural killer (NK) cells, invariant natural killer T (iNKT) cells, gamma delta T (γδ T), mucosal-associated invariant T (MAIT) cells, and macrophages (Mφs). In this review, we describe iPSC-based allogeneic cell therapy, the different culture methods of generating iPSC-derived immune cells (e.g., iPSC-T, iPSC-NK, iPSC-iNKT, iPSC-γδT, iPSC-MAIT and iPSC-Mφ), as well as the recent advances in iPSC-T and iPSC-NK cell therapies, particularly in combinations with CAR-engineering. We also discuss the current challenges and the future perspectives in this field towards the foreseeable applications of iPSC-based immune therapy.

## 1. Introduction

**Figure 1:**
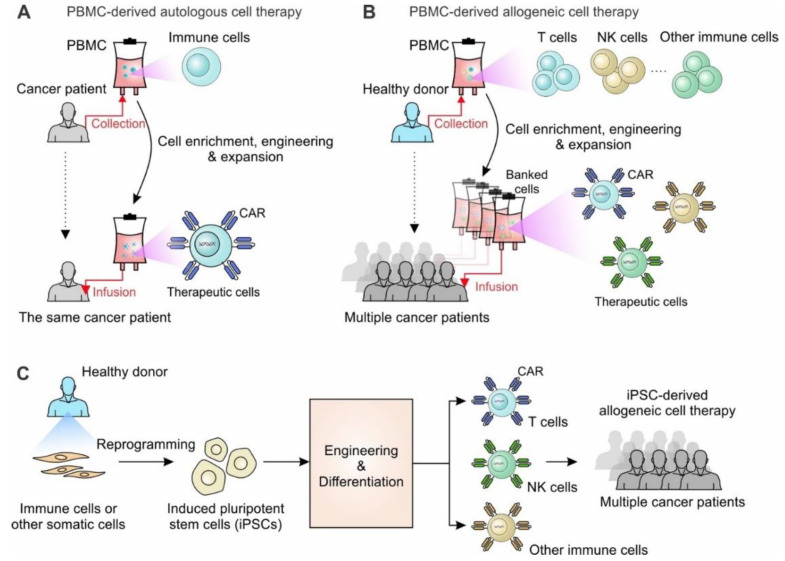
Development of cell therapy from autologous to allogeneic cell therapy. (**A**) PBMC-derived autologous cell therapy. Immune cells collected from cancer patients through leukapheresis are expanded and engineered ex vivo. The engineered cells are infused back to the same patients to fight against cancer. (**B**) PBMC-derived allogeneic cell therapy. Immune cells such as T cells, NK cells and other immune cells are collected from healthy volunteers and stored as banked cells after cell engineering and expansion. The banked cells are ready-to-use and can be utilized to treat multiple cancer patients. (**C**) iPSC-derived allogeneic cell therapy. Immune cells or other somatic cells are collected from healthy donors and reprogrammed to be stable iPSC lines. The reprogrammed iPSCs can be engineered and differentiated into different immune cells for treating multiple cancer patients.

During the past decade, the field of oncology has experienced a remarkable therapeutic overhaul with the advent of cancer immunotherapy, broadening the scope of what cancer therapy can entail. Anti-CTLA-4 monoclonal antibody (mAb) and anti-PD-1 mAb are examples of immune checkpoint blockade (ICB) medications that have shown striking therapeutic efficacy [1,2,3,4,5,6]. Alternatively, adoptive cell therapy, where patients receive autologous immune cells, has also shown promising effects on treating persistent viral infections [7,8,9,10] and malignancies [11,12,13]. In currently ongoing trials of adoptive T-cells therapies, T cells are obtained through leukapheresis from the patient and reinjected back to the patient following ex vivo cell engineering or expansion (Figure 1A). Transferring ex vivo-expanded tumor-infiltrating lymphocytes (TILs) [14,15,16,17] and transferring antigen-specific TCR genes [18,19,20], such as the NY-ESO1 TCR gene [21,22], into engineered peripheral T cells yielded positive results in controlling melanoma [15,21] and other types of tumors [11,16], upon infusion back into the patient. Arming T cells with the expression of a chimeric antigen receptor (CAR) that can specifically target tumor associated antigens (TAAs) has been demonstrated to be extremely effective in the treatment of B-cell leukemia/lymphoma [23,24,25]. The synthetic CAR composes of an extracellular antigen-recognition domain, mostly a single chain variable fragment (scFv), a transmembrane domain and intracellular signaling domains. The extracellular domain was rationally designed to broaden the spectrum of targeted TAAs, including CD19, BCMA, CD22, CD20, EGFR, mesothelin, and many more, and modifications to the intracellular domains resulted in new generations of CAR T cells [26,27,28,29]. Currently, the US Food and Drug Administration (FDA) has already approved six CAR T-cell therapies, axicabtagene ciloleucel (Yescarta, Kite Pharma, 2017), tisagenlecleucel (Kymriah, Novartis, 2017), brexucabtagene autoleucel (Tecartus, Kite Pharma, 2020), lisocabtagene maraleucel (Breyanzi, Bristol Myers Squibb, 2021), idecabtagene vicleucel (Abecma, Bristol Myers Squibb, 2021), and ciltacabtagene autoleucel (Carvykti, Legend, 2022) for treating B cell lymphoma or multiple myeloma patients. However, there are challenges that need to be resolved within the autologous treatment settings: (1) time; (2) cost; (3) heterogeneous quality of therapeutic cells. Therefore, new methods for rapidly producing unlimited antigen-specific T cells with optimized therapeutic features are in high demand and would greatly advance the delivery and efficacy of T cell therapy.

Different strategies have been explored for developing allogeneic cell therapy (Table 1).

One approach is to develop allogenic T cell products, where functional T cells are collected from healthy volunteers rather than from cancer patients who have had exposure to chemotherapy (Figure 1B). The biggest challenge of allogenic T cell therapy is the graft versus host disease (GvHD) risk [30,31]. To avoid immunological rejection and minimize GvHD in patients, allogeneic T cells have to be engineered to remove their human leukocyte antigen (HLA) class I and II molecules as well as disrupt TCR expression [32,33]. These additional genetic modifications increase the tumorigenicity risk and largely decrease the yield of final products. Alternatively, natural killer (NK) cells, which are essential effector cells in the innate immune system, possess features that can circumvent certain challenges in T cell therapies [34,35]. NK cells have shown powerful anti-tumor efficacy in both mice and humans. Unlike T cells that recognize antigens presented by major histocompatibility complex (MHC) molecules, NK cells do not require HLA matching so they are significantly less likely cause GvHD and thus can be utilized as an appropriate cell type for allogeneic cell therapy [36,37]. Because of these potential benefits, NK cells obtained from diverse sources, such as the NK-92 cell line, peripheral blood, and umbilical cord blood, have been evaluated to treat cancer patients in clinical studies [38,39]. Alternatively, unconventional T cells, such as invariant natural killer T (iNKT), gamma delta T (γδT), and mucosal-associated invariant T (MAIT) cells, that naturally do not cause GvHD, have also been investigated as carriers for allogeneic cell therapy [40,41,42]. However, these innate or innate-like immune cells are difficult to be massively expanded, especially after CAR-engineering or other genetic modifications (Table 2).

Induced pluripotent stem cells (iPSCs) have been recognized as an ideal source of engineered off-the-shelf allogeneic cell therapies due to their unlimited expansion capabilities, relative ease of genetic engineering, capacity for clonal selection after genetic modification, and the elimination of the need to collect cells from a donor at any point in time, are recognized as an ideal source for generating off-the-shelf allogeneic cell therapy [43,44] (Figure 1C). Since the discovery that a simple cocktail of transcription factors could restore the pluripotency of adult somatic cells by the Yamanaka group in 2006, enormous progress has been made in the fields of stem cell biology and regenerative medicine [45,46]. iPSCs share similar characteristics with human embryonic stem cells (hESCs) in terms of gene expression and pluripotency, but lack the ethical and regulatory roadblocks to collection that slow progress in hESC research [43,47]. With the introduction of iPSC technology, laboratories all over the world have generated protocols to differentiate various cells of interest. The application of iPSC technology to generate antigen-specific T cell therapy first took hold in Japan during the early 2010s [48,49]. By transducing iPSCs with a CAR or transgenic TCR gene, iPSCs can be converted into antigen-specific T cells with impressive on-target killing efficacies both in vitro and in vivo [48,49,50]. This process can be further optimized for efficacy and safety with the implementation of CRISPR-Cas9 technology for the insertion of CAR genes into endogenous TCRα constant (TRAC) locus chain position, which would reduce the risk of allo-reaction in response to the host [51]. iPSCs innovators, such as the Kaufman group, have also seen substantial success in the derivation of homogeneous, functional NK cells that can be produced at a clinical scale [52,53,54]. In particular, the ‘spin embryoid body (EB)’ protocol together with defined culture conditions makes it feasible to eliminate the use of serum-containing media and stromal cells, producing iPSC-derived immune cells that are better suited for clinical applications. Additionally, unconventional T cells [55,56] and macrophages [57,58] that can also be differentiated from iPSCs are alternative cell carriers for cancer therapy. In this review, we describe the different culture methods of generating iPSC-derived immune cell-based allogeneic therapy, including iPSC-T, iPSC-NK, iPSC-derived unconventional T cells and macrophages, as well as the recent significant breakthroughs, limitations, and future steps towards iPSC cell-based off-the-shelf cell immunotherapy.

## 2. iPSC Technology: Advances and Opportunities

In 2006, the discovery of induced pluripotent stem cells (iPSCs) was heralded as a significant breakthrough in science and medicine. Pioneered by Shinya Yamanaka’s lab in Kyoto, scientists deemed a cocktail of four transcription factors with the ability to induce a shift in somatic cells back to a pluripotent state (OCT4, SOC2, KLF4 and MYC) ‘Yamanaka factors’ [59,60]. The unique cells generated from somatic cells exhibit similar characteristics to embryonic stem cells (ESCs) with equivalent gene expression, indefinite self-renewal capacity, and potential to differentiate into specialized cell types derived from any primary germ layer: ectoderm, endoderm, and mesoderm [45,46,47]. Since the advent of iPSC technology, enormous progress has been made to produce therapeutic cells in the fields of stem cell biology and regenerative medicine. In particular, human iPSC technology, which has rapidly progressed since 2007, was quickly employed to generate human ‘disease in a dish’, implicated in medication screening for drug efficacy and possible toxicity [45,46]. Their human origin, ease of access, expandability, ability to give rise to different cell types, avoidance of ethical concerns related to hESCs, and the possibility to generate personalized medicine utilizing patient-specific iPSCs make them outstanding resources for drug development and understanding disease progression.

There are four major approaches to deliver reprogramming factors: integrating viral systems (e.g., retrovirus, lentivirus), non-integrating vectors (e.g., sendai virus, adenovirus), self-excising vectors (e.g., PiggyBac transposon) and non-integrating non-viral vectors (e.g., CHIR99021, siRNA, and protein delivery) [45,46]. Among them, sendai virus has been widely used as the most efficient and convenient tool. There is a significant effort to establish iPSC banks across the world; however, several requirements need to be fulfilled before banking an iPSC line. According to the International Stem Cell Banking initiative’s recommendations [10,13], most biobanks incorporate the following assessments for establishing iPSC lines: (1) morphology evaluation, (v2) pluripotency assessment, (3) in vitro and in vivo differentiation potential test, (4) transgene silencing examination, (5) karyotype analysis for chromosomal abnormalities, (6) identity confirmation, and (7) microbiological assays avoidance of contamination. To date, several iPSC banks have been established, such as California Institute for Regenerative Medicine (CIRM) in the United States, Center for iPS Cell Research and Application (CiRA) in Japan, and European Bank for induced pluripotent Stem Cells (EBiSC) in Europe [10,13]. These non-profit biobanks obtain samples, generate, and bank clinical-grade iPSC lines to establish a source of standardized, accessible iPSCs across the scientific community. The discovery of iPSC technology not only revolutionized our understanding of cell development, but also opened the door to human-specific drug screening [43,44,45,46]. The rapid advance of iPSC banks opens opportunities for researchers to access these vital cells for both foundational and clinical research.

## 3. Engineering iPSCs for T Cell-Based Therapy

One key component of the adaptive immune system are cytotoxic T lymphocytes (CTLs), which can recognize and kill infected cells and malignant host cells. The innovation of CAR-engineered T cells has resulted in groundbreaking new therapies that can exploit this natural aspect of the body’s immune system for targeted therapies against an array of diseases. CARs are genetically modified synthetic receptors with a single chain variable fragment (scFv) joined to the heavy and light chain variable regions of a specialized monoclonal antibody (mAb) linked by a transmembrane domain directly connected to the intracellular signaling domains [61,62]. CARs drive lymphocytes, primarily T cells, to recognize and kill cells that express the specific antigen. The transduced T cell is endowed with an antibody-like specificity capable of effectively transmitting the intracellular signals needed for T-cell activation. CAR attachment to cell surface antigens occurs independently of the MHC molecules, leading to robust T cell activation and potent anti-tumor responses without any need for co-stimulation. Currently, there are more than 1000 CAR-T cell related clinical trials.

However, autologous CAR-T cell therapy necessitates a bespoke manufacturing method for each patient. Despite the excellent clinical results yielded to date, it has a few well-known drawbacks. The high processing cost, risk of manufacturing failure in some patients, and weeks-long manufacturing processes all result in a delay in treatment availability and make it an infusible treatment option for some patients [12,25,30,61,62]. Patients with highly proliferative diseases, such as acute leukemia, may experience disease progression before their CAR-T product is ready for use, or may lose eligibility due to the disease or other treatments [40,41]. Moreover, patients with T cell dysfunctions, which are characterized in many malignancies due to the immunosuppressive tumor microenvironments, may not have the necessary immunological infracture for an effective autologous T cell response. The preceding lines of treatment also have a deleterious impact on the biological features of autologous T cells. On top of this, the high expense of this sophisticated therapeutic strategy remains a huge burden for the health care system. Thus, generating ‘off-the-shelf’ allogeneic CAR-T cells that allows patients to get treatments right away is in great demand [12,40,41]. This would simplify the process into a single cell product as well as standardizes the CAR-T cell production, allowing redosing if necessary.

### 3.1. Engineering T-iPSCs

**Figure 2:**
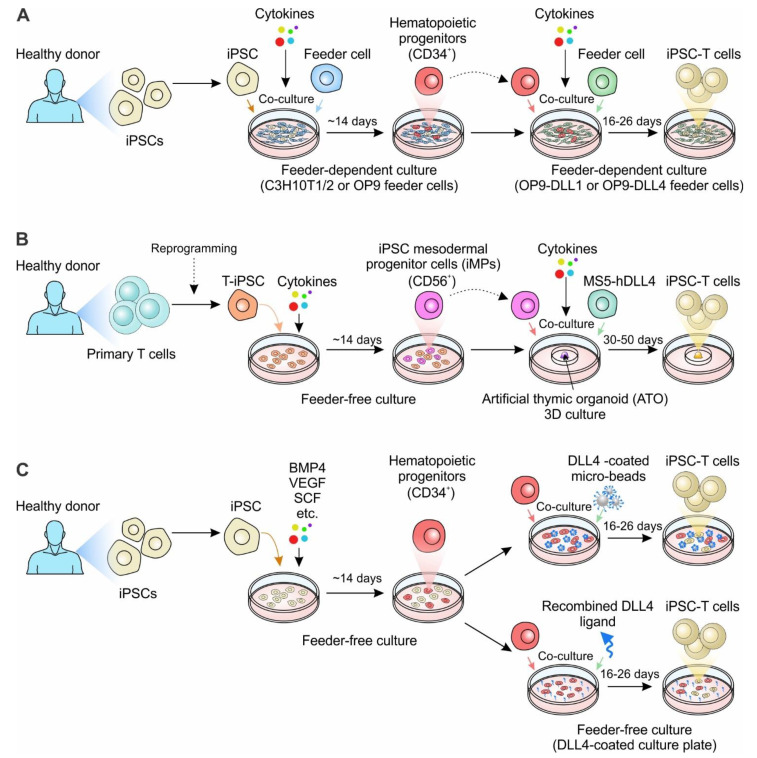
Generation of iPSC-derived T cell-based cell therapy. (**A**) The classic approach to generate iPSC-derived T cells. iPSCs derived from healthy donors are co-cultured with the murine bone marrow stromal cell line C3H10T1/2 or OP9 to allow for the generation of CD34^+^ hematopoietic progenitors. CD34^+^ hematopoietic progenitors are then enriched and co-cultured with OP9 overexpressing DLL1 or DLL4 (OP9-DLL1 or OP9-DLL4) with defined cytokines, driving the differentiation of T cells. (**B**) 3D-organoid culture to generate iPSC-derived T cells. The human primary T cells derived from healthy donors are reprogrammed to iPSCs. The T cell-derived iPSCs (T-iPSCs) are cultured in the defined cytokine cocktail to induce iPSC mesodermal progenitor cells (iMPs). The iMPs are then aggregated with mouse stromal cell line MS5 overexpressing human DLL4 (MS5-DLL4) in the air-liquid interface of the artificial thymic organoid (ATO). (**C**) Feeder-free culture systems to generate iPSC-derived T cells. iPSCs are cultured in the medium with defined cytokines for two weeks to generate hematopoietic progenitors. The CD34^+^ progenitors are selected and reseeded either in the plate containing DLL4-coated micro-beads or plate coated with recombined DLL4 ligand. Notably, for the generation of gene-engineered iPSC-T cells, the healthy donor-derived iPSCs can be replated with genetically-engineered iPSCs, such as CAR-engineered iPSCs.

One approach to massively producing cytotoxic cells is using iPSCs derived from T cells (Figure 2). The rationale behind this method posits that since T cell-derived iPSCs should have inherited the rearranged TCR genes, all regenerated T cells should express the same TCR as the original [63,64]. Since iPSCs can be expanded infinitely, it will be feasible to manufacture as many ‘fresh’ T cells of a given type as needed. Generation of T cells from iPSCs involves a complicated process, requiring first the induction of mesoderm specification and hematopoietic commitment, followed by T cell differentiation [48,50,64,65]. The initial stages of specification to generate hematopoietic lineages were similar among reports. The mesoderm induction is initiated by generating embryoid bodies and/or co-culture iPSCs on murine bone marrow derived stroma cell lines (typically OP9) with morphogens, such as bone morphogenetic protein 4 (BMP4), vascular endothelial growth factor (VEGF), and fibroblast growth factor (FGF), that support mesoderm specification [48,49,50] (Figure 2A). Subsequently, cells are transferred to cytokine cocktails that specifically support hemato-endothelial specification; the resulting CD34^+^ hematopoietic stem cells are collected and replated on a bone marrow-derived stromal cell line OP9 ectopically expressing the Notch ligand Delta-like 1 (DLL1) or DLL4 (OP9-DLL1 and OP9-DLL4) for T cell development [48,50].

The RIKEN and CiRA group in Japan were the first to explore the idea of reprogramming antigen-specific T cells to regenerate clonal cytotoxic T cells [48,49,50] (Table 3). A study by the Kawamoto group generated iPSCs from a MART-1-specific T cell line, the JKF6 cell line, which were long-term cultured tumor infiltrating cytotoxic T cells originally derived from a melanoma patient [48]. JKF6 cells recognize the melanoma epitope MART-1 presented by HLA-A*02:01. MART1-T-iPSCs (iPSCs derived from MART1-specific T cells) were generated by transducing JKF6 with Yamanaka factors [48]. The clones were verified to carry the rearrangement status of the original TCRβ chain, exhibiting hESC-like morphology, as well as expressing pluripotent markers [48]. The MART-1-iPSCs were then cultured with OP9 and subsequently with OP9-DLL1 stromal cells for T cell differentiation. MART-1-iPSCs were successfully developed following DN to DP differentiation. At the DP stage, cells were stimulated with anti-CD3 antibodies for SP differentiation. The MART-1-iPSC-derived CD8^+^ SP T cells largely bear the TCRα chain gene as the original MART-1-iPSCs and are functionally mature with a substantial amount of IFN-γ production responding to peptide stimulation, but lacked the same cytotoxicity as primary T cells [48]. In the same issue of *Cell: Stem Cell*, Nakauchi et al. published their success in growing viral antigen-specific cytotoxic T lymphocytes (CTLs) by adding to the RIKEN’s approach [49]. An HIV type 1 (HIV-1) epitope-specific T cell was selected and induced into iPSC cells. These HIV-1-T-iPSCs (iPSCs from HIV-1-specific T cells) were then re-differented into rejuvenated T cells that showed the same pattern of TCR gene arrangement as the original T cells [49]. Similarly, the in vitro differentiation included two steps: iPSCs giving rise to mesoderm-derived hematopoietic stem cells and/or progenitor cells, and T cell differentiation from hematopoietic stem cells. Instead of using OP9 cells at the beginning, as in Kawamoto’s paper, the T-iPSCs generated by Nakauchi et al. were cocultured on C3H10T1/2 feeder cells in the medium supplemented with VEGF, stem cell factor (SCF), and FMS-like tyrosine kinase 3 ligand (FLT-3L) and then on day 14, cells were transferred to OP9-DL1 co-culture in the presence of FLT-3L and interleukin-7 (IL-7) [48,49]. These too, had reduced efficacy, and lacked the antigen-specific cytotoxicity that was expected of adaptive immune cells.

Using T-iPSCs (T cell-derived iPSCs) as base cells, the reprogrammed iPSC clones can inherit the original TCR and drive the re-differentiation into iPSC-T (T cells re-differentiated from iPSCs) cells. However, this method requires time-consuming cloning of antigen-specific T cells and is limited to antigens that can be identified from patient-specific T cells. Moreover, the therapeutic application of iPSC-T cells is restricted in that the recipient patients need to match the HLA of their donors, greatly limiting the ‘universality’ of any off-the-shelf applications [64,66]. CAR-engineering, however, redirects T-cell specificity in an HLA-independent manner, expelling the need of HLA restriction and enhancing anti-tumor properties. In a 2013 paper by Themeli et al., researchers generated T-iPSC clones by reprogramming peripheral T cells from a healthy donor, then transduced a second-generation CAR specific for CD19 into the selected T-iPSC clone [50]. The CAR-expressing iPSC-T cells exhibited potent anti-tumor efficacy in a xenograft model but were phenotypically similar to innate γδT cells [50]. This method generated an innate type T cell with the expression of a CD8αα homodimer, impacting the re-differentiated T cels’ antigen-specific cytotoxic capacity in a similar manner to MART-1-specific T cells. The conventional method was then modified with Maeda et al.’s purification DP cells, which were then stimulated them with monoclonal anti-CD3 antibodies (Abs) to generate CD8αβ T cells that exhibited comparable antigen-specific cytotoxicity to the original CTLs [65].

More recently, a 3D organoid culture system was reported to successfully generate CAR T cells for ‘off-the-shelf’ manufacturing strategies [69] (Figure 2B). In Wang et al., iPSC clones were reprogrammed from primary CD62L^+^ T cells, the naïve and memory T cell population which was recognized to have exceptional persistence and enhanced clinical outcomes in CAR-T cell therapy [69]. T-iPSC clones were transduced with lentivirus encoding a CD19-targeting CAR (19CAR). Instead of utilizing the OP9 system, the 19CAR^+^ T-iPSC clones were sorted and cultured under feeder-free conditions to simulate mesodermal development [69]. Then CD56^+^CD326^−^ iPSC mesodermal progenitor cells (iMPs) were sorted and mixed with a mouse stromal cell line overexpressing DLL4 (MS5-hDLL4) feeder cells to differentiate to hematopoietic progenitors, followed by T cell differentiation for additional 5–7 weeks [69]. Unlike iPSC-T cells generated from the monolayer coculture systems displaying an innate-like phenotype, the regenerated 19CAR iPSC T cells from the 3D organoid culture system showed comparable phenotypes to conventional T cells and exhibited similarly potent antitumor function [69].

The classic approaches to generate iPSC-derived T cells requiring feeder cells in each stage, such as OP9 and MS5 cells, have paved the way to allogeneic therapy (Table 1). However, using murine-derived stroma feeder layers increases the risk of cross-species contamination [67,68]. Feeder cell maintenance relies on different serum and basal media, making it challenging for quality control. Developing feeder-free and serum-free culture systems is important for broadening the application of iPSC-T-based ‘off-the-shelf’ therapy (Figure 2C). In Iriguchi et al. 2021, researchers provided notch signaling for T cell differentiation by incorporating immobilized delta-like 4 (DL4) protein together with retronectin in place of feeder cells [67] (Table 1). The results demonstrated the feasibility of generating progenitor T cells in a feeder-cell-free condition. Alternatively, DL4-μbeads, along with cytokine cocktails, can also induce an ordered sequence of T cell differentiation [68]. All these approaches provide a simple and robust platform for producing clinically applicable T cells, as well as for studying human T- cell differentiation [67,68].

### 3.2. Engineering iPSCs in Combination with TCR Transgene Overexpression

Despite promising results from using T-cell-derived iPSCs (T-iPSCs) to generate CTLs, certain issues remain limiting the establishment of highly potent T-iPSCs, such as low reprogramming efficiency of T cells into iPSCs and heterogeneity among T-iPSC clones. To overcome these challenges, researchers developed an alternative method where iPSCs were transduced with exogenous TCR genes and then re-differentiated the TCR-engineered iPSCs into CTLs [63,65,66]. With this approach, it was much easier to establish high-quality TCR-iPSCs clones with guaranteed specificity and quality using TCR and iPSCs genes. This method has been verified on different non-T-cell-derived iPSCs, such as myeloid cell-, monocyte- and fibroblast-derived iPSCs, to successfully generate T cells through the transduction of exogenous TCR genes [64,70]. In particular, the cells regenerated from the HLA-homo iPSCs could be transplanted into HLA-hetero patients with minimal rejection. Additionally, this approach has been utilized for treating viral infectious diseases (e.g., COVID-19) [64]. The model has also been tested in the feeder-free culture system to generate WT1-T cells that were specific to the Wilms tumor 1 (WT1) as well as CD19 CAR T cells [67,68].

The combined power of somatic cell reprogramming, CAR-engineering, and gene-editing technology can be used to produce cytotoxic T cells with antigen specificity mimicking that of the human adaptive immune system (Figure 2 and Table 1). Additional modifications such as genetic ablation of HLA expression could be utilized to reduce allogeneic response against these cells and prevent GvHD [51]. Overexpressing minimally polymorphic HLA-E molecules in HLA-deletion T cells can resolve NK lysis of HLA absent cells [51]. Moreover, additional modifications such as the deletion of inhibitory receptors (e.g., PD-1) can further improve anti-tumor activity [51]. With these advances, we should eventually reach the goal of using human iPSC-derived T cells as a novel, standardized anti-cancer therapy that can utilize the evolved potency of the human adaptive immune system.

## 4. Engineering iPSCs for NK Cell-Based Therapy

### 4.1. NK Cells as a Promising Alternative to T Cells for Cellular Therapy

Natural killer (NK) cells are innate cytotoxic lymphocytes that target malignant cells through degranulation. NK cells can also orchestrate an immune response through the release of cytokines. Unlike T cells, which need to be primed against a specific antigen, NK cells can directly kill damaged cells based on the summation of inhibitory and activating signals received from their germline-coded surface receptors. Killer-cell immunoglobulin-like receptors (KIRs) are a class of inhibitory NK receptors that will bind to the major histocompatibility complex class I (MHC I) on adjacent cells and pass on an inhibitory signal. Cells that don’t have the correct MHC I expression will lack the inhibitory KIR-MHC I ligation signal, allowing NK cells to recognize non-self-cells. Conversely, NK cell-activating receptors bind to stress-induced ligands that are expressed during conditions such as DNA damage or hypoxia [71]. The binding of those ligands allows for recognition of cells that are in altered states, allowing NK cells to kill them through degranulation. NK cells are also able to kill opsonized cells through CD16 recognition of the IgG Fc region through a process called antibody-dependent cellular cytotoxicity (ADCC) [72,73,74]. Altogether, these characteristics allow NK cells to respond to a variety of diseased cell states, including cancerous cells.

NK cells have been proven to be safe and effective against certain cancers in various adoptive cell therapy studies without causing GvHD, and engineering only adds to their virulence [71,75,76,77,78,79]. NK cells are a prime candidate for genetically engineered CAR therapies because they are significantly less likely to cause complications such as cytokine release syndrome (CRS), neurotoxicity, or GvHD; they do not require HLA matching, which increases their potential for off-the-shelf allogeneic therapies; and they have multiple mechanisms by which they are able to activate cytotoxic effects independent of the CAR [72,74,75,80,81,82]. For these reasons, many are shifting their focus from CAR-T therapies to CAR-NK therapies. Currently, there are many ongoing CAR-NK phase I/II clinical trials targeting both hematological malignancies such as acute lymphocytic leukemia (ALL) and non-Hodgkin’s lymphoma (NHL), and solid tumor cancers such as colorectal and pancreatic cancer. One such hematological malignancy study from Liu et al. demonstrated the effects of anti-CD19 CAR NK cells in patients with relapsed or refracted CD19^+^ B cell malignancies. NK cells were isolated and expanded from cord blood then transduced using a retroviral vector containing genes for anti-CD19 CAR, membrane-bound IL-15, and inducible caspase 9. Of the 11 patients treated, 8 responded to treatment, 7 of which patients were able to achieve a complete remission with no reports of serious or irreversible toxicities. The caspase 9 kill switch was not activated at any point during the study because no patients exhibited severe adverse side effects [83] (Clinicaltrials.gov Identifier NCT03056339). CAR-NK therapies have been less effective in certain cancers due to poor trafficking, infiltration, and persistence in vivo, so additional gene engineering has been employed to create “armored” CAR-NK cells that have increased fitness [74,84]. Adding a transgene for membrane-bound IL-15 allows for longer persistence of CAR-NKs in vivo and alleviates the risk of CRS that comes with repeated cytokine injections needed for stimulation [85]. Knocking out the CISH gene deletes the cytokine-inducible Src homology 2-containing (CIS) protein, a negative regulator of IL-15 signaling, which increases the cell’s metabolic activity and subsequent anti-tumor activity [84,86]. These and many other studies show that allogeneic CAR-NK cells are both safe to use in clinical applications and can be effective in cancer treatments.

### 4.2. Generation of iPSC-NK Cells

**Figure 3:**
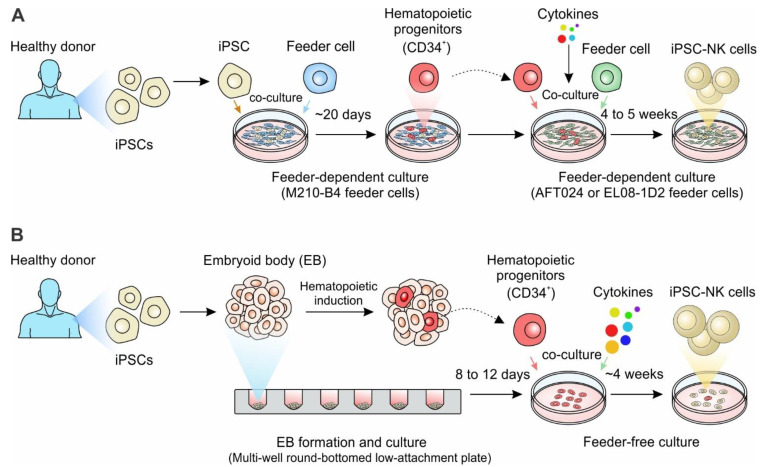
Generation of iPSC-derived NK cell-based cell therapy. (**A**) The iPSCs derived from healthy donors are co-cultured with the murine bone marrow stromal cell line M210-B4 for ~20 days to allow for the generation of CD34 + hematopoietic progenitors. CD34 + hematopoietic progenitors are then enriched and co-cultured with a monolayer of murine AFT024 (a fetal liver-derived stromal cell line) or EL08-1D2 stroma cells with defined cytokines for 4 to 5 weeks, eventually generating mature NK cells. (**B**) The iPSC derived from healthy donors are spun to aggregate in a multi-well round-bottomed low-attachment plate, forming the embryoid bodies (EBs) of uniform size in each well. After 8 to12 days of culture, the hematopoietic progenitor cells containing EBs were transferred to feeder-free plates in NK differentiation media containing cytokine combinations for 4 weeks to generate iPSC-derived NK cells. Notably, for the generation of gene-engineered iPSC-NK cells, the healthy donor-derived iPSCs can be replaced with genetically-engineered iPSCs, such as CAR-engineered iPSCs.

Like iPSC-Ts, iPSCs are a renewable source that can be used to generate homogenous NK cell populations for off-the-shelf allogeneic therapies [74,80,85] (Figure 3). Methods for generating NK cells from iPSCs were first adapted from the human embryonic stem cell NK (hESC-NK) differentiation protocols, since hESCs were the main source of stem cells before the discovery of iPSCs [71,87]. The original two-step feeder-dependent culture method initially outlined by the Kaufman group entailed coculturing iPSCs with mouse stromal cell line M210-B4 for 19-21 days to induce hematopoietic stem cell (HSC) differentiation [88,89] (Figure 3A). The CD34^+^ CD45^+^ hematopoietic progenitors were isolated and then cultured on a second murine stromal line AFT024 or EL08-1D2 with a cocktail of cytokines including IL-3, IL-7, IL-15, SCF, and Flt-3L for 4–5 weeks to generate mature NK cells [80,88,89] (Figure 3A). This method produced functional, mature NK cells that demonstrated the ability to potently kill multiple types of tumor cells. However, from a large-scale manufacturing perspective, maintaining murine-derived feeder cells requires additional cost and increases the risk of cross-species contamination [53,71,74,90].

Subsequently, the Kaufman group developed an embryoid body (EB)-derived feeder-free 3D culture method (Figure 3B). iPSCs were initially grown under feeder-free conditions for a week, then aggregated into ultra-low attachment 96-well plates to form EBs [91]. Cells within EBs form self-stromal cells to help support the growth of lymphocytes, which eliminates the need for other stromal cell lines. EBs were cultured in the feeder-free media supplemented with SCF, BMP4, VEGF to induce the formation of HSC progenitors. After 8-12 days of culture, the hematopoietic progenitor cells containing EBs were transferred to feeder-free media containing IL-3, IL-7, IL-15, SCF, and Flt3L for 4 weeks to generate CD45^+^CD56^+^ NK cells [53,92]. The iPSC-derived NK cells can be further expanded using different NK culture methods and share similar growth rate, phenotypes, and activity compared to PB-NK cells and UCB-NK cells [53,71,73,74,89].

### 4.3. iPSC-NK Cells in Cell Therapy

iPSCs provide an ideal platform for NK-based allogeneic therapy. Once the stable engineered iPSC lines are established, they can be indefinitely expanded and used to produce a standardized population of appropriately engineered iPSC-derived NK cells. Preclinically, many studies are highlighting the efficacy of engineered iPS-NK cells against cancers. In a representative study, iPSCs have been genetically engineered to express 3rd generation CARs, which express a single-chain antibody fragment recognizing either CD19 or mesothelin, a CD8α hinge region, the transmembrane protein CD28, a co-stimulatory protein 4-1BB, and the activating domain CD3ζ. NK cells derived from this dual CAR-engineered iPSC exhibited higher killing abilities towards CD19^+^ or mesothelin^+^ tumor cells [93]. This can further be improved by the knockout of the IL-15 signaling regulatory protein CISH, creating iPS-NK cells with improved metabolic profile, increased expansion and persistence, and enhanced cytotoxicity in human AML xenograft tumor models. Additionally, the deletion of CISH does not affect the pluripotency or stability of iPSCs. A separate study from the Kaufman group showed that CARs generated against the NK-activating domain NKG2D greatly improves the anti-tumor activity in iPS-NKs by activating PLC-gamma, the Syk-vav1-Erk pathway, and the NK-κB pathway. As a result, these iPS-NKG2D-CAR-NK cells show improved granulation, cytokine production, and cytotoxicity towards antigen-expressing tumor cells [94].

Clinically, there are several universal off-the-shelf iPSC-derived NK cell products currently undergoing Phase I trials: FT500, FT516, FT576, and FT596, all of which are products of Fate Therapeutics (Clinicaltrials.gov Identifier NCT03841110, NCT04023071, NCT05182073, NCT04245722). To date, these are the only iPSC-derived NK therapy products cleared for use in clinical investigations within the United States. The most modified of these four trial therapies is FT576, which is an iPS-NK therapy engineered with a B cell maturation antigen (BCMA) CAR for use in patients with multiple myeloma (MM; Clinicatrials.gov Identifier NCT05182073). BCMA-CAR recognizes BCMA, an antigen highly expressed on malignant plasma cells, which allows for tumor recognition. This antigen-directed killing is further improved by the addition of hnCD16 Fc receptors, which help augment the effect of ADCC, and an IL-15 fusion receptor (IL-15RF), which enhances NK activity, as well as supplementation with daratumumab, which is an anti-CD38 monoclonal antibody that prevents the possibility of NK cell fratricide [95]. In their preclinical studies, FT576 NK cells outperformed peripheral blood NK (PB NK) cells in vitro during fratricide and cytotoxicity assays and were found to have greater persistence than PB NK cells. When using FT576 alongside daratumumab in MM mice models, FT576 was able to achieve complete clearance [96]. FT576 is a promising cell therapy candidate with the possibility to become the first curative MM therapeutic, but clinical results have yet to be seen.

Overall, iPSC-NK cells have shown to be a promising alternative to T cells for cell immunotherapy due to their ability to be genetically engineered, cultured at a large scale, and their adaptation to treating various cancers. However, the in vivo persistence and the durability of iPSC-NK cells, as well as their efficacy in combination with other immune checkpoint inhibitors are still unclear and remain to be clarified in order to move the iPSC-derived NK cells to clinical applications for the treatment of hematologic and solid tumors.

## 5. Engineering iPSCs for Other Immune Cell-Based Therapy

### 5.1. iPSC-Engineered Macrophage Cell Therapy

Macrophages (Mφs) constitute heterogeneous populations of immune cells that play plastic and vital roles in embryonic development, tissue homeostasis and innate immunity [97]. In the context of host immune response, ‘classically activated’ M1 Mφs, typically induced by lipopolysaccharide (LPS), interferon (IFN)-γ, or tumor necrosis factor (TNF), provide rapid “first-line” defense upon infection by direct engulfment and elimination of pathogens in peripheral tissues [98,99]. Furthermore, Mφs secrete pro-inflammatory cytokines and serve as professional antigen-presenting cells that process and present foreign antigens to T lymphocytes, bridging the innate and the adaptive arms of the host immune response [100]. In contrast, ‘alternatively activated’ Mφs or M2 Mφs, that are induced by interleukin (IL)-4 and IL-13 can produce anti-inflammatory cytokines to attenuate inflammation, mediating wound healing and tissue homeostasis [99,101]. This ability to alternate between activation states in response to microenvironmental cues has made Mφs attractive targets for the mitigation of many diseases related to immune function [99]. Dysregulation of Mφs may result in uncontrolled and chronic inflammation and has been implicated in the pathogenesis of chronic inflammation and autoimmune diseases [102,103,104]. On the contrary, immune suppression induced by anti-inflammatory Mφs has been shown to mediate fibrosis and promote tumor survival, growth, and metastasis [99,105]. Since tumor-associated Mφ (TAM) infiltration of solid tumors is frequently associated with poor prognosis and chemotherapy resistance, much effort has been dedicated to eliminating or re-differentiating TAMs into a pro-inflammatory state to remediate the tumorsuppressive effects of the tumor mircroenvironment (TME) [105,106,107].

Given the effective innate response of Mφs and their capacity to infiltrate solid tumors, Mφs have been genetically engineered with CARs and tested for their therapeutic relevance in both hematologic malignancies and solid tumor models [108]. Klichinsky et al. reported successful generation of human anti-CD19- and anti-HER2-CAR-macrophages (CAR-Ms) targeting myelogenous leukemia and ovarian cancer, respectively [108]. These CAR-Ms exhibit a pro-inflammatory (M1) phenotype and can induce pro-tumor M2 Mφs into anti-tumor M1 Mφs [108]. In human xenograft mouse models, CAR-Ms recruit and activate cytotoxic T lymphocytes, which in turn leads to a pro-inflammatory tumor microenvironment and suppression of tumor growth [108]. This translational research further led to the first-in-human trial of CAR-Ms, CT-0508, and is currently enrolling patients (Clinicatrials.gov Identifier NCT04660929). Preliminary data from the Phase 1 clinical trial showed that anti-HER2 CAR-Ms effectively target and suppress HER-2 positive solid tumors with high safety profiles (Clinicatrials.gov Identifier NCT04660929). Promising results are indicating that macrophage-based cell therapy is a novel pathway toward cancer immunotherapy, however, unlike CAR-T cells that can expand robustly upon activation, CAR-Ms proliferate poorly both in vitro and in vivo [57,109]. Therefore, a more scalable method of CAR-M manufacture is needed to fully unleash its therapeutic potential. Traditional methods used to generate Mφs in vitro include: (1) direct isolation of tissue-resident macrophages (TRMs) from human tissue [110]; (2) establishment of immortalized human Mφ cell lines (such as THP-1 and U937) [111]; and (3) generation of monocytes-derived macrophages (MDMs) [110]. However, direct isolation of TRMs is limited by the scarcity of available human tissues, while immortalized human Mφ cell lines and MDMs lack biological relevance, to varying extents, and do not fully recapitulate TRMs [110].

iPSC-derived macrophages may provide a feasible solution for the large-scale production of readily distributable CAR-Ms. Various detailed protocols that are used to differentiate iPSC-derived Mφs have been extensively discussed in a well-written review by Lyadova et al. [110]. Embryoid body formation is the most commonly used method to differentiate iPSC-derived Mφs, and specific modifications include rotary suspension [112], microwell approach [113], microfluidic hanging drop chip [114], and others. Upon formation of iPSC-derived EB, myeloid Mφs are further induced by supplementation of cytokines, including GM-CSF, M-CSF, IL-3, and IL-4 [115]. In addition to EB formation, iPSC-derived macrophages have also been obtained through the mesoderm-hemogenic endothelial (HE)-hematopoietic stem and progenitor cell (HSPC) axis in a feeder-free system [116]. Although iPSC-Mφ differentiation through HSPCs resulted in more phenotypically defined progenitors, which can serve as valuable models to study Mφ development in vitro, it requires large amounts of cytokines, which makes large-scale commercialization difficult. Encouragingly, Ackermann et al. developed industry-compatible bioreactor-based mass production of human iPSC-Mφ in a feeder-free suspension system, taking a step forward to the commercialization of iPSC- Mφs [117]. Zhang et al. first attempted generation of iPSC-derived CAR-Ms (CAR-iMac) by first reprogramming PBMCs back to iPSC using non-integrative episomal vectors (encoding OCT4, SOX2, KLF4, LIN28, and L-MYC), which were engineered with antigen-specific CARs by lentiviral transduction to create CAR-iPSC clones [57]. CAR-iPSCs were subject to EB formation and cultured in APEL II medium supplemented with BMP-4, bFGF, VEGF, and SCF for hematopoietic specification, and substitution of BMP-4 with IGF-1, IL-3, M-CSF, and GM-CSF at day 8 was used for myeloid lineage differentiation [57]. These CAR-iMac cells exerted phagocytosis against tumor cell lines such as K562 and OVCAR3 in an antigen-specific manner, namely through CD19-CAR and mesothelin-CAR, respectively [57]. However, further investigation is required to demonstrate whether iPSC-derived CAR-iMac can also recruit and activate donor-derived T cells and/or endogenous T cells to promote anti-tumor immunity, as seen in CAR-engineered primary macrophages [57,108].

### 5.2. iPSC-Engineered MAIT Cell Therapy

Mucosal-associated invariant T (MAIT) cells are unconventional innate-like αβT lymphocytes expressing a semi-invariant T cell antigen receptor (TCR) (TRAV1-2-TRAJ33 in both humans and mice, and less frequently TRAV1-2-TRAJ12 or TRAV1-2-TRAJ20 in humans) [118,119]. MAIT cells are less abundant in mice compared to NKT cells, but they are more frequent in humans, comprising approximately 1–10% of peripheral T cells and 20–50% of T cells in the liver [118]. MAIT cell TCR-dependent activation is achieved through TCR recognition of microbial-derived vitamin B2 (riboflavin) metabolites presented by a monomorphic MHC class I-related molecule, MR1 [119]. While MAIT cells are well known for their important role in host immune response against microbial and viral infections, emerging evidence has suggested their involvement in human cancer-related immune response [118,120]. The frequency of circulating MAIT cells was decreased in patients with colorectal cancer (CRC), lung cancer, kidney cancer, brain cancer, and hepatocellular carcinoma (HCC) [118]. This corresponds to increased infiltration of MAIT cells into tumor tissues, as was observed in patients with kidney cancer, brain cancer, and CRC [118,121]. Mechanistically, MAIT cells exhibit solid tumor-infiltrating capacity at least in part through increased expression of T cell-trafficking chemokine receptors, such as CCR6 and CXCR6, in patients with CRC [121]. Functional assays show that MAIT cells induce direct anti-tumor cytotoxicity both in vitro and in vivo [121,122]. However, seemingly contradictory results exist in patients with HCC where high infiltration of MAIT cells correlates with unfavorable clinical outcomes [123,124]. This can possibly be explained by the observation that tumor-infiltrating MAIT cells showed an exhausted phenotype, with high-level expression of CTLA-4, PD-1, and TIM3 [123,124]. These results imply that MAIT cells may be potential therapeutic targets for treating human malignancies, either through eliminating pro-tumor MAIT cell subtypes or genetically engineering human MAIT cells towards a tumor-targeting effector state. However, the exact underlying mechanisms by which MAIT cells play opposing roles in the context of human cancer pathogenesis, or initiate crosstalk with other immune cell types, remain elusive and require further investigation.

MAIT cells have become the subject of increasing interest for genetically engineered CAR-T cell cancer immunotherapy due to their innate-like effector properties, chemotherapy resistance, and low risk of GvHD upon allogeneic cell transfer [125]. MAIT cells express high levels of IL-12 receptor (IL-12R), IL-18R, and IL-23R, enabling inflammatory cytokine-induced TCR-independent activation. MAIT cells also express activating NK cell receptors such as NKG2D and CD161, which are known to interact with their corresponding ligands expressed on tumor cells [126]. Thus, MAIT cells are capable of achieving tumor recognition and activation through alternative mechanisms other than detection of MR1-presented antigen [126]. This potentially renders MAIT cells more resistant to tumor antigen escape commonly observed in CAR-T treated cancer patients suffering from relapse. Upon activation, MAIT cells produce anti-tumor cytokines (IFN-γ and TNF-α), and cytotoxic molecules such as perforin and granzyme-B [126]. Moreover, most MAIT cells are at G_0_ phase and express multidrug resistance transporter (MDR/ABCB1), enabling MAIT cells to be more resistant to chemotherapy in comparison to other T cell populations [127]. Furthermore, owing to their semi-invariant TCRs, MAIT cells protect recipient mice from acute GvHD following bone marrow transplantation, and do not mediate alloreactivity [120,128,129]. All these characteristics suggest MAIT cells as a promising candidate for developing “off-the-shelf” allogeneic CAR-MAIT cell therapy. However, to the best of our knowledge, comprehensive functional studies on CAR-MAIT cells in suppressing tumor growth have not been reported, though we expect more functional studies related to CAR-MAIT cell therapy will soon appear due to recent advances of MR1 tetramers [130].

iPSCs potentially provide an unlimited source of CAR-MAIT cells for readily distributable allogeneic cell therapy [50]. Wakao et al. first reported the successful generation of MAIT-iPSC-derived MAIT-like lymphocytes expressing definitive MAIT cell surface markers TCR Vα7.2, CD161, and IL-18R [131]. To first establish MAIT-iPSC clones, MAIT cells were purified from fresh cord blood (CB) using anti-Vα7.2 monoclonal antibody and transduced with Sendai viral vector carrying reprogramming factors KLF4, SOX2, OCT-3/4, and C-MYC [131]. Re-differentiation of MAIT-like lymphocytes from MAIT-iPSC was achieved by co-culturing with OP9-DLL1 [131]. After the 30-day co-culturing, more than 98% of the lymphocytes were TCR Vα7.2^+^ with a majority expressing CD161 and IL18R [131]. Although these MAIT-like lymphocytes showed similar global gene expression profiles to that of CB-MAIT cells, it remains unclear whether these MAIT-like lymphocytes retain anti-tumor specificity through MR1-recognition and effective tumor-killing cytotoxicity. To date, ex vivo generation of iPSC-derived CAR-engineered MAIT cells remains theoretical. Further investigations are required to test whether it is feasible to construct iPSC clones that are reprogrammed from more standardized somatic cell sources such as fibroblasts or peripheral blood T cells and are capable of re-differentiating into human MAIT cells.

### 5.3. iPSC-Engineered γδ T Cell Therapy

γδ T cells represent a unique T cell subtype expressing a T cell antigen receptor (TCR) composed of a γ chain and a δ chain, first identified in 1987 [132,133]. Since then, significant efforts have been invested in elucidating the enigmatic roles of these innate-like T lymphocytes in human physiology. γδ T cells are regarded as “unconventional” T cells because they are a minor population of the T cell lineage, comprising around 1–5% of circulating CD3^+^ lymphocytes in most adult animals [134]. Human γδ T cells exhibit limited diversity of TCR composition, with Vδ1, Vδ2, Vδ3, and Vγ2, Vγ3, Vγ4, Vγ5, Vγ8, Vγ9, Vγ11 representing the most common gene segments used during TCR δ and γ chain rearrangement, respectively [135]. In contrast to conventional αβ T cells, γδ T cells are not restricted to recognition of peptide-derived antigens presented by MHC molecules. Instead, the Vγ9Vδ2 TCR is activated upon engagement with phosphorylated antigens presented on butyrophilin (such as BTN3A1 and BTN2A1) molecules, although the exact mode of interaction remains largely unknown and is under intensive investigation [136].

Following the original discovery that there are increased numbers of γδ T cells present in patients with multiple myeloma (MM) treated with aminobisphosphonates (such as Zoledronate, ZOL) [137], various studies have been conducted to evaluate the immunotherapeutic relevance of γδ T cells [138]. γδ T cells are observed in high frequency in multiple types of cancer [136], and, upon TCR-dependent activation, they induce rapid and effective target cell killing through the secretion of pro-inflammatory cytokines (IL-2, IL-12, IL-15, IL-18, etc.) and cytotoxic molecules (granzymes and perforin) [138]. In view of the HLA independence of γδ T cells and relatively limited diversity of their TCR rearrangements, γδ T cells do not elicit GvHD upon allogeneic cell transfer [139]. In addition to the TCR-dependent activation by phospho-antigens, γδ T cells also express activating NK cell receptors, such as NKG2D, DNAM-1, NKp30, and/or NKp44, and show potent cytotoxicity against stressed, abnormal cells, particularly malignant cells [140,141,142]. There is further evidence showing that γδ T cells are the initial source of anti-tumor cytokine IFN-γ [143], and serve as professional APCs [144], which may in turn recruit and regulate activation of tumor-specific αβ T cells, acting synergistically towards effective anti-tumor immunity. Moreover, unlike αβ T cells, γδ T cells are less frequently found in secondary lymphoid organs and preferentially migrate to peripheral tissues (most commonly barrier surfaces like the skin, intestine, lung, etc.) [145]. This potentially renders γδ T cells a crucial role in tumor immunosurveillance and may be the basis for their capacity to infiltrate solid tumors. Due to their potent anti-tumor cytotoxicity, immune-regulatory functions, homing and infiltration capacity towards peripheral tissues, as well as safe immunogenicity profiles, γδ T cells have attracted increasing interest in the development of allogeneic cell-based cancer immunotherapy [134,136,138]. In September 2021, GammaDelta Therapeutics announced the initiation of a first-in-human phase 1 trial (GDX012) of allogeneic, non-engineered γδ T cells for treating acute myeloid leukemia (Clinicatrials.gov Identifier NCT05001451). As the current clinical trials start to unveil the anti-tumor potential of “off-the-shelf” allogeneic γδ T cell therapy in clinical patients, further genetic modifications with tumor-targeting CARs or administration in combination with immune checkpoint blockades may further benefit the therapeutic outcomes of γδ T cells in the context of human cancer.

During the past decade, established evidence has shown that a majority of γδ T cells in the peripheral blood express Vγ9Vδ2 TCRs [146], and functional studies suggest Vγ9Vδ2 T cells are the most potent effector γδ T populations [136]. Vγ9Vδ2 T cells target and kill a variety of solid tumors and lymphoma/leukemia cells in vitro and in vivo [136,138,146], and thus, further discussion will mainly focus on Vγ9Vδ2 T cells, unless otherwise noted. Building upon that, several groups have reported successful generation and evaluation of CAR-engineered γδ T cells in suppressing tumor growth both in vitro and in vivo [147,148,149]. Deniger et al. were among the first to generate engineered γδ T cells with bispecific tumor targeting, namely through Vγ9Vδ2 TCR and anti-CD19 CAR [147]. Anti-CD19-CAR-γδ T cells were ex vivo expanded with CD19-expressing artificial APC and showed enhanced killing against CD19-positive tumor cells lines both in vitro and in vivo, in comparison to γδ T cells without CAR engineering [147,150]. More recently, a group led by Herrman M. from Adicet Therapeutics developed anti-Glypican-3 (GPC-3) CAR-γδ T cells, equipped with constitutively secreted IL-15. In vitro and in vivo killing assays demonstrated that these cell products effectively target and kill hepatocellular carcinoma (HCC) cell lines with prolonged persistence [148] (note, Adicet Therapeutics uses δ1T cells). Furthermore, Nishimoto et al. from Adicet Therapeutics isolated, expanded, and engineered PBMC-derived γδ T cells with anti-CD20 CAR [149]. These CD20-CAR-γδ T cells exhibited both innate (rapid secretion of pro-inflammatory cytokines) and adaptive (TCR-dependent tumor cell killing) anti-tumor response against B cell lymphoma both in vitro and in xenograft immunodeficient mice [149]. These remarkable advances in proof-of-concept investigation have led to the clinical trial of ADI-001 from Adicet Therapeutics in treating B cell non-Hodgkin’s lymphoma with anti-CD20-CAR-γδ T cells, which is currently recruiting patients (Clinicatrials.gov Identifier NCT04735471).

Although there has been a great number of encouraging results in recent years, there are some drawbacks to utilizing γδ T cells as CAR-T therapy carriers. Due to the fact that γδ T cells only constitute a small proportion (1–5%) of PB T lymphocytes, current protocols actively used to generate autologous and/or allogeneic γδ T cell therapy rely heavily on ex vivo expansion with ZOL and pro-inflammatory cytokines such as IL-2 and IL-15 [137,148,149]. However, ex vivo expanded T cells gradually lose their anti-tumor potential due to T-cell exhaustion brought on by long-term stimulation, which is also known as T cell exhaustion [151]. Thus, there is an unmet need to manufacture homogeneous and functional CAR-engineered γδ T cells with standardized quality controls on a large scale. To circumvent this roadblock, several groups have investigated whether iPSCs can serve as an unlimited source of γδ T cells. Watanabe et al. reported the generation of γδ T cells from human γδ T cell-derived iPSCs [152]. The researchers first activated PBMC-derived γδ T cells with ZOL and IL-2, and subsequently transduced them with Sendai viral vector to re-program γδ T cell-derived iPSC clones [152]. Around 70% of the resultant iPSC clones expressed TCRs rearranged from TCRG and TCRD gene locus [152], indicating retention of the original TCR genes. Next, γδ T cell-derived iPSCs were cultured in Stem Fit medium on laminin-511 E8 fragments, as previously described [153], and two iPSC cell lines were able to differentiate. Whether they were able to generate promising results, such as hematovascular precursor markers and CD34^+^CD43^+^ expression in the majority of cells, however, the group did not investigate whether it is feasible to generate functional and phenotypically defined γδ T-like cells from these HSPC populations. Functional studies are required to further evaluate the anti-tumor capacity of γδ T cells generated from these iPSC clones. More recently, Zeng et al. showed that mimetic γδ T cells can be generated from γδ T cell-derived iPSCs and skewed towards more NK-like effector cells (designated as γδ natural killer T cells, γδ NKT cells) [154]. These γδ NKT cells express an array of NK-activating receptors such as NKG2D, NKp30, NKp44, NKp46 and DNAM-1, with low to no expression of inhibitory killer immunoglobulin-like receptors (KIRs) [154]. Similar to previous reports, the group isolated Vγ9Vδ2 T cells from PBMC and transduced them with Sendai viruses carrying the Yamanaka reprogramming factors. However, this did not result in viable iPSC clones that can be seeded onto feeder fibroblasts [154]. As alternatives, they tried using episomal vectors derived through nucleofection, which resulted in iPSC cell lines derived from γδ T cells [154]. Interestingly, to obtain iPSC-derived γδ T-like cells with increased NK functions, the group co-cultured the γδ T-iPSC clones with OP9-DLL1 feeder cells under NK cell “promoting” differentiation conditions previously developed in their lab [154]. Through in vitro tumor-killing assays with antibody blockage of different surface receptors, γδ NKT cells were shown to exhibit effective tumor cell cytotoxicity via NK activating receptors as well as CD16-dependent ADCC [154].

### 5.4. iPSC-Engineered iNKT Cell Therapy

Invariant natural killer T (iNKT) cells, also known as type I or classical NKT cells, are a subpopulation of unconventional T cells. They express an invariant TCR chain V14-J18 that is paired with V8/7/2 in mice or V24-J18 with V11 in humans [155,156]. Unlike conventional T cells that recognize peptides presented by MHC molecules, iNKT cells recognize glycolipids such as alpha-galactosylceramide (α-GalCer) presented by the MHC-I-like molecule CD1d [157]. iNKT cells are at the forefront of a variety of immunological responses by secreting a wide range of cytokines in response to lipid antigen stimulation and serving as a bridge between innate and adaptive immune systems [158]. More importantly, iNKT cells do not recognize mismatched MHC molecules, freeing them from causing GvHD. Multiple clinical trials have reported that the adoptive transfer of iNKT cells was associated with reduced GvHD [159]. However, the extremely low number of iNKT cells in peripheral blood (0.01–1% of PBMCs) restricts iNKT cell-based immunotherapy. We have recently developed different approaches to generate iNKT cells from engineered hematopoietic stem cells (HSCs) using either the bone marrow-liver-thymus (BLT) mouse model or artificial thymic organoid (ATO) culture system [160,161,162,163]. The HSC-derived iNKT cells represented similar characteristics compared to PBMC-derived iNKT cells with enhanced NK functions against cancers [160,161]. The unique innate feature of iNKT cells and their capability to bridge innate and adaptive immunity have cultivated an increasing interest in generating iNKT-based cell therapy. Using a combination of iPSC technology, iNKT cells may play a significant role as an ideal allogeneic cell carrier for next-generation medicine.

The first step in the generation of effective iNKT-based cell therapy was the in vitro differentiation of fibroblasts into iNKT cells by Watarai et al. [164]. The differentiation was achieved using the OP9/DLL1 culture system with the addition of cytokine combinations such as IL-15 and IL-7. The generated iPSC-iNKT cells expanded in response to α-GalCer stimulation and produced abundant Th1 cytokine INF-γ, induced dendritic cell maturation, and activated both CTL and NK cells [164]. Using the same culture system, Kitayama et. al. reported that human iNKT cell-derived iPSCs can re-differentiate into functional iNKT in vitro [56]. The generated human iPSC-iNKT cells not only recapitulated the adjuvant effect of natural iNKT cells, but also exhibited NK cell-like cytotoxicity against cancer cell lines [56]. These preclinical studies provide foundations for studying human iNKT cell biology and the clinical translation of allogeneic iPSC-iNKT cell therapy.

In October 2020, Professor Motohashi at Chiba University Hospital led the World’s first clinical trial of iPSC-derived NKT cells for head and neck cancer [165]. This study was done in an allogeneic situation, where NKT cells, isolated from a healthy donor, were re-programmed to iPSCs and further differentiated into iPS-NKT cells in vitro. The expanded NKTs are administered 3 times every 2 weeks to the blood vessels of cancer patients and are intended to activate the patient’s immunity in an antigen-agnostic manner, similar to how an adjuvant works. This study may provide supporting information to translate allogeneic iPSC-iNKT cell immunotherapy to clinical applications.

## 6. Outlook

While autologous CAR-T cells have shown promising therapeutic results in patients with relapsed or refractory B-cell malignancies and multiple myeloma, the current technology has several safety and logistical flaws, emphasizing the need to explore alternative populations for cellular treatment, particularly allogeneic cell therapy (Figure 1A,B). iPSCs are promising cell sources for generating off-the-shelf therapy (Figure 1C). However, despite the breakthrough in differentiating iPSCs into T, NK or other immune cells, there are still challenges to be overcome and necessary concerns to address.

Cell therapy, especially off-the-shelf therapy, requires high yield of final products for transplantation. Current iPSC differentiation culture approach can already generate approximately 600 × 10^6^ of iPSC-T cells and 10^5^-fold of iPS-NK cells from 1 × 10^6^ of iPSCs for example, which are applicable for iPSC-based cell therapy [67,68,69]. The current classic culture approaches to iPSCs re-differentiation are largely dependent on murine-derived feeder cells, which can increase the risk of cross-species contamination. The utilization of serum for maintaining feeder cells may also increase the variation of final cell products, presenting major issues for large-scale industrial standardization and real-patient applications and clinical trials. A few studies have started to develop xeno-free and feeder-free culture systems that are more suitable for industrialized applications [67,68]. However, before these can be brought to large-scale production it must be determined: (1) How these approaches can be applied to a wide array of products? (2) Whether they hold up under the scrutiny of quality control standards at a large scale? (3) Whether the yield large enough for multiple patients?

In addition to the evaluation of these xeon-free culture techniques, the extent to which the T, NK, macrophages, and other cells derived from iPSCs truly resemble the original immune cells must be determined before they can safely be introduced into patients at a large scale. Papers like that of Kawamoto et al. found that a small portion of T cells aberrantly expressing TCRαβ was found during the DP stage [44,45,46]. It was explained that during the TCRA-encoded a chain assembly stage, the negative-feedback regulation for a could be loosely compared to that for b chain, resulting in a small portion of T cells expressing rearranged TCRα. Although the number was low, such rearranged TCRα was exceedingly undesirable that could potentially convert the tropism of TCR, making re-differentiated T cells incapable of attacking targeted antigens, and, more importantly, might cause GvHD in patients. By mimicking TCR signaling using anti-CD3 antibodies during the DN-to-DP transition stage or alternatively, CRISPR knockout of a recombinase gene in the T-iPSCs, could end the expression of RAG genes or prevent undesired additional TCR assembly [65,66]. The aberrantly early expression of TCRαβ from pre-assembled TCRα and TCRβ in murine thymocytes could lead to subsequent lymphomagenesis, which raises concerns about the safety of T-iPSCs [166]. As a result, the redifferentiation approach needs to be further refined and clinically validated before being used in real-world treatments, and the employment of an inducible suicide-gene system to eliminate undesired malignancies might be necessary.

The heterogenicity of final products may be important for the therapeutic outcome. One of the benefits of using iPSCs is the flexibility to select a specific phenotype of immune cells before reprograming them into a stable iPSC line. The progeny cells, for example, the T-iPSC derived T cells, can inherit certain epigenetic modifications from their original immune cells and recapitulate the heterogeneity in the final product. However, the iPSC-derived CAR T cells were found to be predominantly CD8-expressing with low levels of MHC [69]. While low MHC expression is desirable to improve iPSC-T persistency by reducing host versus graft (HvG) response, the imbalance between CD4^+^ and CD8^+^ populations may be a bottleneck for the application [69]. It is still challenging to generated CD4^+^ T cells with current in vitro culture methods, which could potentially be resolved by manipulating the culture conditions or additional gene editing.

Moreover, iPSCs represent the “primed” state, and are heterogeneous in both cell population and differentiation potential. iPSC clones show large variations in differentiation efficiency. The tumorigenicity and toxicity of iPSCs pose a significant safety risk of iPSC-derived immune cell therapy [167]. Instead of using reto/lenti-virus to reprogram iPSCs, using episomal vectors, Sendai virus vectors or modified RNAs that do not result in chromosomal integration provides a safer way to mitigate the tumorigenicity and toxicity [167]. Removing undifferentiated cells or optimizing culture methods to generate pure final cell product is also necessary. Regardless, the redifferentiation approach needs to be further refined and clinically validated before being used in real-world treatments, and the employment of an inducible suicide-gene system to eliminate undesired malignancies might be necessary. However, what type of cells should be considered as good starting materials for iPSC-based therapy? How robust are the current differentiation approaches for generating clinically scalable numbers of cell products? CRISPR/CAS9-based genome-wide genetic modification and CAR-engineering could open a new avenue for utilizing human iPSCs, but will these modifications impact the quality or quantity of final products? What is the most efficient way to better manage the off-target risk of gene modifications? All these questions remain to be better clarified. It is important that final products be carefully selected and thoroughly characterized prior to clinical application in order to protect the safety of patients and maximize the efficacy of the medicines being developed.

Besides, the iPSC-generated cell therapeutics may still encounter obstacles in treating solid tumors, which is mainly due to the intrinsic barriers imposed by the hostile immunosuppressive tumor microenvironment (TME), including the formation of extracellular matrix (ECM) derived from cancer-associated fibroblasts (CAFs), anti-inflammatory cytokines secreted by suppressive immune cells (Tregs, tumor-associated macrophages, as well as myeloid-derived suppressive cells), and competition for metabolic fuels which limits long-term immune cell persistence [168,169,170]. The TME impedes therapeutical cells trafficking, infiltration, persistence, or function in the immunosuppressive milieu by producing suppressive soluble factors and by overexpressing negative immune checkpoints [171]. To overcome these hurdles, a series of genetical engineering approaches have been used to manipulate the chemotaxis and tissue homing of therapeutical cells for improving trafficking and infiltration, or to design new CARs such as tumor stroma targeting for depletion of immunosuppressive cells or depletion of stromal cells at TME. To counteract immune cell dysfunction in solid tumors, checkpoint (CTLA-4, PD-1 and TIM-3) blockades have also been tested in combination of cell therapies [172]. Alternatively, pro-inflammatory cytokine-armored immune cells, such as IL-12-, IL-18- or IL-23-armored T cells were tested to re-shape TME to favor T cell anti-tumor immunity [168,173,174]. Likewise, similar gene-engineering strategies can be applied to modify the iPSCs before differentiating into final products, which could pave the way towards the generation of “off-the-shelf” iPSC-derived cells for an adoptive cell therapy (ACT) with a broader therapeutical spectrum.

The advent and adoption of iPSC technology represent a paradigm shift in CAR-based call therapy, revolutionizing the potential for universal ‘off-the-shelf’ therapy from unlimited cell supplies. While there is a significant amount of research that must be done to address the questions and concerns regarding the safety and efficacy of iPSC-generated cell therapies, we already foresee its irreplaceable role in developing next-generation medicines. We look forward to more studies and clinical trial results coming out in the next few decades that will push the field forward and improve the life-saving capabilities of cell-based immunotherapy.

## Figures and Tables

**Table 1:** Current allogeneic cell therapies in clinical trials.

Clinical Trial	Description	Cell Product	Malignancies	Company
NCT03841110	FT500 in combination with checkpoint inhibitors against solid tumors	iPSC-NK	Solid tumor	Fate Pharmaceutics
NCT04630769, NCT04023071	FT516 and IL2 with Enoblituzumab for ovarian cancer; FT516 combination with CD20-directed monoclonal antibodies	iPSC-NK (non-cleavable CD16 Fc receptor)	Ovarian cancer; Advanced B-cell lymphoma	Fate Pharmaceutics
NCT04555811; NCT04245722	FT596 with Rituximab as relapse prevention after autologous HSCT for NHL; FT596 as a monotherapy and in combination with anti-CD20 monoclonal antibodies	iPSC-NK(hnCD16, IL15RF, CAR-19)	B cell lymphoma (BCL); Chronic lymphocytic leukemia (CLL)	Fate Pharmaceutics
NCT04714372; NCT05069935; NCT04614636	FT538 in combination with Daratumumab in acute myeloid leukemia (AML); FT538 in combination with monoclonal antibodies in advance solid tumors; FT538 in subjects with advanced hematologic malignancies	iPSC-NK (hnCD16, IL15RF + CD38KO)	AML, MM, solid tumors	Fate Pharmaceutics
NCT05182073	FT576 in subjects with multiple myeloma (MM)	iPSC-NK (hnCD16, IL15RF + CD38KO, CAR-BCMA)	MM	Fate Pharmaceutics
NCT04629729	FT819 in subjects with B-cell malignancies	iPSC-T (CAR-19, TCR-KO)	BCL, CLL, ALL	Fate Pharmaceutics
NCT03190278	Study evaluating safety and efficacy of UCART123	Allogeneic T-cells expressing anti-CD123 CAR	Relapsed/refractory acute myeloid leukemia (AML)	Cellectis
NCAT041500497	Phase 1 study of UCART22 in patients with R/R CD22+ BALL	Allogeneic T cells expressing anti-CD22 CAR	Relapsed or refractory CD22 + B-cell acute lymphoblastic leukemia	Cellectis
NCT04142619	Study evaluating safety of and efficacy of UCART targeting CS1 in patients with R/R MM	Allogeneic T cells expressing anti-CS1 CAR	Relapsed/refractory MM	Cellectis
NCT02735083; NCT02808442; NCT02746952	Study of UCART19 in patients with R/R BALL	Allogeneic T cells expressing anti-CD19 CAR	R/R BALL	Cellectis
NCT04416984	Safety and efficacy of ALLO-501A anti-CD19 allogeneic CAR T cels in adults with R/R LBCL	Allogeneic T cells expressing anti-CD19 CAR, CD52 KO, TCR KO	R/R LBCL, R/R NHL	Cellectis/Allogene
NCT04093596	Safety and efficacy of ALLO-715 BCMA allogeneic T cells in adults with R/R MM	Allogeneic T cells expressing anti-BCMA CAR, CD52 KO, TCR KO	R/R MM	Cellectis/Allogene
NCT04696731	Safety and efficacy of ALLO-316 in subjects with advanced or metastatic clear cell RCC	Allogeneic T cells targeting CD70	RCC	Allogene
NCT04991948; NCT03692429	Study of Pembrolizumab treatment after CYAD-101 with FOLFOX reconditioning in mCRC	Allogeneic T cells targeting NKG2DL	mCRC	Celyad
NCT04613557	Safety, activity and cell kinetics of CYAD-211 in patients with R/R MM	Allogeneic T cells targeting BCMA	r/r MM	Celyad
NCT03769467; NCT04554914; NCT03394365; NCT02822495; NCT03131934; NCT01192464	Therapeutic effects of Tebelecleucel in subjects with diseases	EBV-CTL (Tabelecleucel, or tab-cel)	EBV-induced lymphomas and other diseases	Atara
NCT03283826	Phase 1/2 study to evaluate the safety and efficacy of ATA188 in subjects with progressive MS	EBV-CTL	Progressive MS	Atara
NCT05252403; NCT03389035	Residual disease driven strategy for CARCIK-CD19 (CMN-005) in adults/pediatric BCP-ALL	Allogeneic CARCIK-CD19	ALL	Coimmune
NCT04735471; NCT04911478	A study of ADI-001 in B cell malignancies	Allogeneic CD20-targeted gd T cells	B cell maliganacy	Adicet Bio

R/R: relapsed or refractory; BALL: B-cell acute lymphoblastic leukemia; LBCL: large B cell lymphoma; NHL: non-Hodgkin lymphoma; KO: knock-out; RCC: renal cell carcinoma; mCRC: metastatic colorectal cancer; EBV: Epstein-Barr virus; NPC: Nasopharyngeal carcinoma; MS: multiple sclerosis; ALL: acute lymphoblastic leukemia.

**Table 2:** Pros and cons of using different types of cells in cancer immunotherapy.

Cell Therapy	Pros	Cons
Conventional T cell	Abundant source Easy to expand in vitro Scalable and standardized quality controls for manufacturing	Time-consuming and costly MHC dependent T cell exhaustion GVHD Low ability of trafficking and infiltrating into solid tumors
NK cell	No need for previous antigen priming Multiple innate activating receptors that can mediate killing MHC independent No GVHD	Low persistence in the absence of cytokine Low number in patients Low ability of trafficking and infiltrating into solid tumors
iNKT cell	Innate and adaptive features Invariant TCR recognizes lipid antigens presented by CD1d No GVHD Low toxicities	Low number in patients May have immunosuppressive properties (Th2, Th17) Limited clinical data with CAR-iNKT cells
γδT cell	Innate and adaptive features MHC independent No GVHD Low toxicities	Extremely low number in patients May have immunosuppressive properties (γδ T17, Vδ1 γδ T cells, γδ Treg) Limited clinical data with CAR- γδT cells
MAIT cell	Solid tumor-infiltrating capacity Direct anti-tumor cytotoxicity both in vitro and in vivo Resistant to tumor antigen escape	Unclear mechanisms in suppressing tumor Lack of clinical data with CAR-MAIT cells
Macrophage cell	Penetration into solid tumors Phagocytosis of tumor cells and innate immune response No GVHD Cross present antigens to αβ T cells	Poor proliferation both in vitro and in vivo May have immunosuppressive properties (M2) Limited clinical data with CAR-macrophage cells

**Table 3:** Representative in vitro differentiation methods of generating iPSC-derived T cells for cancer cell therapy.

Publications	Final Products (Immune Cell Types)	Start Material	iPSC Genetic Modification	Feeder or Feeder-Free	Overall Procedure Time	Major Components in Culture Medium
Nishimura et al., 2013 [49]	Conventional αβ T cells	CD3^+^ PBMC T cells for a healthy donor; HIV-1-specific CD8^+^ T cells	NA	Feeder: C3H10T1/2, OP9-DL1	33–40 days	VEGF, SCF, FLT-3L, IL-7, IL-15
Themeli et al., 2013 [50]	Conventional αβ T cells	Peripheral blood T lymphocytes (PBL) from a healthy donor	19CAR-engineering	Half feeder-free; Half feeder: OP9-DLL1	~30 days	BMP-4, FGF, VEGF, SCF, FLT-3L, IL-3, IL-7
Vizcardo et al., 2013 [48]	Conventional αβ T cells	JKF6 cells (MART-1 specific TILs)	NA	Feeder: OP9, OP9-DLL1	~40 days	SCF, FLT-3L, IL-7, IL-2
Maeda et al., 2016 [65]	Conventional αβ T cells	LMP2-specific CTLs from a healthy donor	NA	Feeder: OP9, OP9-DLL1	~6–8 weeks	IL-7, FLT-3L, SCF, IL-2, IL-21
Minagawa et al., 2018 [66]	Conventional αβ T cells	GPC3-specific CTLs from GPC3 peptide-vaccinated patients; Monocyte-derived HLA-typed iPSCs	*RAG2* knockout; WT1-TCR transduction	Feeder: C3H10T1/2, OP9-DLL1	NA	FGF, VEGF, SCF, IL-7, FLT-3L, IL-15,
Maeda et al., 2020 [63]	Conventional αβ T cells	Monocytes derived iPSCs from the HLA-homo donor	WT1-TCR transduction	Feeder: OP9, OP9-DLL1	~36 days	FGF, IL-7, FLT-3L, SCF, IL-7, IL-21
Iriguchi et al., 2021 [67]	Conventional αβ T cells	Peripheral blood T cells; HIV-1-specific CTLs; RAG2-deleted GPC3 T-iPSCs	NA	Feeder-free	~42 days	CHIR99021, BMP4, FGF, VEGF, SCF, TPO, FLT-3L, SDF1α
Trotman-Grant et al., 2021 [68]	Conventional αβ T cells	Human IPS11- and STIPS cell lines	NA	Feeder-free	~42 days	BMP4, FGF, VEGF, FLT-3L, SCF, IL-7
Wang et al., 2021 [51]	Conventional αβ T cells	Human iPSC line: GPC3-16-1 (generated from CTLs)	*PVR* knockout; HLA-E transduction; *B2M* knockout; *CIITA* knockout	Feeder: OP9-DLL1	NA	CHIR99201, FGF, VEGF, BMP4, SCF, FLT-3L, TPO, IL-7, IL-15
Wang et al., 2022 [69]	Conventional αβ T cells	iPSC clones from CD62L^+^ T cells	19CAR-engineering	Feeder: MS5-DLL4	~51–64 days	BMP4, VEGF, FGF, EGM-2, SB-431542, SCF, FLT3, IL-7, TPO, IL-2, IL-7

LMP2: Latent membrane protein 2; CTLs: cytotoxic T lymphocytes; GPC3: Glypican 3; RAG2: recombinase-activating-gene-2; NA: not available.
